# The bumblebee *Bombus terrestris* carries a primary inoculum of *Tomato brown rugose fruit virus* contributing to disease spread in tomatoes

**DOI:** 10.1371/journal.pone.0210871

**Published:** 2019-01-17

**Authors:** Naama Levitzky, Elisheva Smith, Oded Lachman, Neta Luria, Yaniv Mizrahi, Helen Bakelman, Noa Sela, Orly Laskar, Elad Milrot, Aviv Dombrovsky

**Affiliations:** 1 Department of Plant Pathology, ARO, The Volcani Center, Rishon LeZion, Israel; 2 The Mina & Everard Goodman Faculty of Life Sciences, Bar-Ilan University, Ramat-Gan, Israel; 3 The Hebrew University of Jerusalem, Agroecology and plant health, Rehovot, Israel; 4 Israel Institute for Biological Research (IIBR), Ness-Ziona, Israel; Universita degli Studi del Piemonte Orientale Amedeo Avogadro, ITALY

## Abstract

The bumblebee *Bombus terrestris* is a beneficial pollinator extensively used in tomato production. Our hypothesis was that bumblebee hives collected from a *Tomato brown rugose fruit virus* (ToBRFV) infected tomato greenhouse, preserve an infectious primary inoculum. Placing a bumblebee hive collected from a ToBRFV contaminated greenhouse, in a glass-/net-house containing only uninfected healthy tomato plants, spread ToBRFV disease. Control uninfected tomato plants grown in a glass-/net-house devoid of any beehive remained uninfected. ToBRFV-contaminated hives carried infectious viral particles as demonstrated in a biological assay on laboratory test plants of virus extracted from hive components. Viral particles isolated from a contaminated hive had a typical tobamovirus morphology observed in transmission electron microscopy. Assembly of ToBRFV genome was achieved by next generation sequencing analysis of RNA adhering to the bumblebee body. Bumblebee dissection showed that ToBRFV was mostly present in the abdomen suggesting viral disease spread via buzz pollination. These results demonstrate that bumblebee hives collected from ToBRFV-contaminated greenhouses carry a primary inoculum that reflects the status of viruses in the growing area. This new mode of ToBRFV spread by pollinators opens an avenue for detection of viruses in a growing area through analysis of the pollinators, as well as emphasizes the need to reevaluate the appropriate disease management protocols.

## Introduction

Tomato production is jeopardized by the two newly discovered tobamoviruses *Tomato mottle mosaic virus* (ToMMV), reported in Brazil [1], Mexico [2] and USA [3], and *Tomato brown rugose fruit virus* (ToBRFV) reported in Jordan [4] and Israel [5].

The outbreak of ToBRFV that has occurred in Israel overcame the *Tm-2*^*2*^ resistance of commercial tomato verities. The disease started in 2014 at Ohad village in Southern Israel and within two years the disease spread to all tomato-growing areas across the country [5]. A recent report on ToMMV infection of commercial tomato verities emphasizes the potential spread of this virus as well [6]. Further studies are required to confirm the capability of ToMMV to overcome the *Tm-2*^*2*^ resistance in commercial tomato verities. ToBRFV, a single stranded positive sense RNA virus that belongs to the *Tobamovirus* genus was first reported in Jordan. ToBRFV has typical rigid rod-like particles that differ in length [5]. The viral genome encompasses at least four open reading frames (ORFs). ORF 1 and ORF 2 together, separated by a leaky stop codon, form the RNA dependent RNA polymerase complex (RdRP). ORF3 encodes the Movement Protein (MP) and the small ORF4 is translated to the Coat Protein (CP). Similar to other tobamovirus species, ToBRFV is efficiently transmitted by mechanical contacts. In commercial tomato greenhouses, the tomato plants are exposed to mechanical transmission of the virus during the agro-technical activities that are performed continually to shape the plants e.g. pruning and trellising.

In tomato production, the bumblebees *Bombus terrestris*, L. (*Hymenoptera*: *Apidae*) that are used extensively around the world for pollination purposes, contribute to fruit quality and quantity [7–10]. In Israel, more than 2,200 hectare of tomato greenhouses are pollinated solely by bumblebees, which are provided by specialized companies during the growing cycle.

The bumblebees have eusocial behavior, characterized by co-operation between sterile female cast (workers) that care for the offspring of fertile female cast (queens) that lay the eggs. Pollination activity is usually held by the workers that use their bodies to shake the anther of the flowering plants by aggressive vibration movements resulting in pollen collection. This type of pollination is known as buzz pollination [11]. The bumblebees are known to excel in buzz pollination probably due to their massive structure. Previous studies have shown that the *Potexvirus Pepino mosaic virus* (PepMV) [12]and two *Pospiviroids*, *Tomato chlorotic dwarf viroid* (TCDVd) and *Tomato apical stunt viroid* (TASVd) [13, 14] are transmitted by bumblebees. One study had shown that while foraging bumblebees are capable to transmit the tobamovirus *Tobacco mosaic virus* (TMV) from infected to un-infected tomato plants grown adjacent to each other in a green-house [15]. However, in that study a primary inoculum had to be already present in the greenhouse in order to achieve bumblebee mediated TMV transmission [15]. The aim of the current study was to investigate the possibility that bumblebees might preserve infectious ToBRFV viral particles in hives collected from virus contaminated greenhouse. Indeed we show that bumblebees from the contaminated hives spread the disease to healthy uninfected elite tomato plants and therefore are considered a primary source of infection of the tobamovirus ToBRFV and potentially of other mechanically transmitted plant viruses.

## Materials and methods

### Bumblebee hives

ToBRFV-contaminated bumblebee hives (26cm long x 22cm wide) were collected from ToBRFV contaminated commercial tomato greenhouses from the HaBsor area Western Negev or Ramat Negev, Israel. Hives were transferred to Volcani Center and served for laboratory and glass-/net-house experiments. Un-exposed bumblebee hives purchased from Bio-Bee biological system Ltd (Sde-Eliyaho) were used as negative controls for the experiments.

### Glasshouse experiment using a ToBRFV-contaminated-beehive (experiment #1)

In a glasshouse (6 m long and 6.3 m wide), 37 tomato seedlings cv. M82 were planted in 10 L pots. Following flowering, on day 10–14 -post-planting, a “used” bumblebee hive containing ~30–40 bumblebees, was transferred from a ToBRFV contaminated commercial tomato greenhouse in the HaBsor area Western Negev, Israel (the hive was ~14 days in the commercial greenhouse), to the experimental glasshouse, for 19 days. The plants were grown for 41 days post planting (dpp) and the produced fruits did not reach maturation. In parallel, in a separate glasshouse, a control experiment was conducted. For that purpose 10 tomato seedlings cv. M82 that were picked from the same source, were grown for the same time period untouched, without any beehive insertion. Leaf samples from all the plants in the experiment were collected individually for ELISA analysis. Hive components, cotton and comb, were analyzed by RT-PCR for the presence of ToBRFV.

#### Large-scale net house experiments (experiments #2 and #3)

Two experiments were conducted sequentially in a 50 mesh-net house (20 m long and 7.8 m wide) at the Volcani Center. Experiments #2 and #3 were carried out in April 2017 and in June 2017, respectively. Tomato seedlings (130 and 128 plants in experiments # 2 and # 3, respectively) were planted in a coconut coir potting mix, and were arranged in rows. In experiment #2 cv. Ikram plants were planted and in experiment #3 cv. Ikram and Shiran were planted.

In experiment #2, on the twentieth day post planting, a “used” bumblebee hive (with 30–40 bees) was placed in the net house. The hive was transferred from ToBRFV contaminated commercial tomato greenhouse from the HaBsor area, the Western Negev, Israel (the hive age was ~14 days in the commercial greenhouse). The hive was then kept in the experiment for 26 days. In experiment #3 on the twenty seventh day post planting, two “used” bumblebee hives (with 10–20 bees each) were placed in the net house. The hives were transferred from ToBRFV contaminated commercial tomato greenhouses in the Ramat Negev area, the Western Negev, Israel (the hive age in the greenhouse was ~30 days). In experiment #3 that was performed on June, bumblebees apparently suffered from the hot temperature leading to early mortality of the bumblebees in the hives and following 14 days in the net house experiment, all were dead. In parallel to experiments #2 and #3, 20 un-infected healthy tomato seedlings that were picked from the same source (planting tray) for each experiment, were grown in a separate net-house lacking any beehive and were kept un-touched for the appropriate duration of the experiments. Leaf samples from all the plants in the experiment were collected individually for ELISA analysis. Hive components, cotton and comb, from experiment # 2 were analyzed by RT-PCR for the presence of ToBRFV.

#### Virus testing by indirect enzyme-linked immunosorbent assay (ELISA)

Samples of plant tissue (100 mg) and bumblebee-hive components (cotton, which surrounds the beehive structure in a way that the bees pass in and out through this isolating cotton element, and comb, which is a structure in the beehive that is built by the queen and workers and used for laying eggs) that were picked arbitrarily from hive elements, were tested by indirect ELISA (using non antibody-coated plates) [16]. Samples were ground in 1 ml coating buffer and were analyzed in duplicates. Laboratory produced ToBRFV antiserum [5] diluted to a ratio of 1:5,000 was added and plates were incubated for 3 h at 37°C (or overnight (ON) at 4°C). A commercial alkaline phosphatase (AP) conjugated goat anti-rabbit (IgG) (Sigma, Steinheim, Germany) was added for 3 h at 37°C (or ON at 4°C). The substrate (p-nitro phenyl phosphate, Sigma) was used at a concentration of 0.6 mg mL^-1^. Plates were recorded by an ELISA reader (Thermo Fisher’s Multiskan FC), at 405 nm and 620 nm. ELISA results were considered positive when the values amounted to a minimum ratio of three times the value of the Negative Reference (3xNR). Samples that showed lower ratios were reexamined by ELISA test and by RT-PCR (see below).

#### Reverse transcription (RT) and PCR amplification

Plant tissue or bumblebee-hive components (cotton, comb, bumblebees and dissected bumblebees) were soaked in a general extraction buffer (3–6 volumes per weight) (Bioreba, Switzerland), ground with mortar and pestle (Bioreba, Switzerland), and the crude lysate served for viral RNA extraction (Bio Neer, South Korea). 10–12 μl of viral extracted RNA were used as a template for reverse transcription (RT) using Reverse Transcriptase (RevertAid, Life Technologies, USA) with the complementary primers designed for ToBRFV genome: R-3’UTR 5’ ACCCCCGGTAGGGGCCCA ‘3 at position 6,392 nt. and R-RdRP 5’ CTAATGCGTCTCCCGACACT ‘3; at position 1,572 nt. The attained viral cDNA served as a template for PCR amplification using JMR PCR mix (JMR Holdings) and ToBRFV specific primers: F-5,476, 5’ GAAGAAGTTGTTGATGAGTTCAT 3’ and R-6,287, 5’ GATTTAAGTGGAGGGAAAAACAC 3’. We have also used ToBRFV specific primers at position 5,931–6,307 encoding the capsid protein and at position 1–1,572 of the 5’ UTR and the region encoding the small subunit of the replicase, as previously described [5]. For detection of the ToBRFV genome that adhered to the bumblebee, RNA was extracted from the washing solution of a washed bumblebee (see below), and subjected to RT-PCR. For reverse transcription, we used Maxima Reverse Transcriptase cDNA kit (Thermo Fisher Scientific) and the reverse primer 5,531R: 5’ TGCAAGCCTTACAGACATCG 3’. For PCR we used Phusion High-Fidelity DNA Polymerase (Thermo Scientific) and the primers F-264: 5’ AGGGCATATCCAGAATTCCA 3’ and R-5,531. PCR amplicons were separated by electrophoresis on a 1% agarose gel and visualized by ethidium bromide staining under UV camera (Bio-Print TX4). For selected amplicons, the bands were excised from the agarose gel using purification column (Bio Neer) and subjected to Sanger sequencing at Hy-Lab (Rehovot, Israel).

### Protein separation by SDS-PAGE and Western blot analysis

50–100 mg of bumblebee-hive components, which included the enveloping cotton, the comb, and the nectar that is stored in the empty comb cells after the grown bees emerge, were suspended in 1ml USB buffer [50 mM Tris-HCl pH 7.0, 8M Urea, 2% SDS and 10 mM β-mercaptoethanol] for 30–40 minutes before homogenization. Then the extracts were boiled at 97°C for 10 minutes followed by centrifugation at 12,000 g for 10 minutes and 100 μl from the supernatant were mixed with 4X Laemmli buffer [17] prior to SDS-PAGE separation. Protein samples from hive components were separated by 15% SDS-PAGE.

SDS-PAGE gels were electro-blotted onto nitrocellulose membranes (for 30 min at 240 mA for two membranes) using a semi-dry transfer blot apparatus (Bio-Rad). Blotted proteins were visualized by staining with Ponceau S staining buffer [0.2% Ponceau S pure, 5% glacial acetic acid and PBS]. After washing the blotted membranes with PBS they were blocked with PBS (pH 7.4) containing 3% non-fat milk powder for 2 h at room temperature (or overnight at 4°C) with slow agitation. An anti ToBRFV antiserum diluted in PBS (1: 2,000) was added to the membranes with slow agitation for ON at 4°C. Following the primary antiserum incubation, the membranes were washed three times with PBS-tween (PBS-T). Then, a commercial AP-conjugated goat anti-rabbit (IgG) (Sigma) was added at 1: 5, 000 dilution ratio and incubated for 1 h at room temperature with shaking. Following washes with PBS-T, the ToBRFV CP was visualized by adding the substrate (NBT& BCIP; Promega).

#### Detection of ToBRFV in dissected tomato flowers

At the flowering stage, infected and un-infected tomato plants were dissected to separate the leaves, sepals, petals and the stamen encompassing the carpel. The plant tissue parts were suspended in equal ratios of μl USB buffer/mg tissue. Homogenized extracts were boiled for 10 min and then subjected to Western blot analysis.

### Virus purification from bumblebee hive components

120 g of comb from beehive B were suspended in 100 ml of 0. 1M phosphate buffer pH 7.0 and 10% chloroform and n-butanol (1:1, v/v), were added. The sample was than stirred and warmed to 37°C for over 2 hr. The comb-wax in the bumblebee hive structure is not prominent therefor the structure disintegrates in a warm solvent and the use of vortex. The sample suspension was centrifuged in a GSA rotor for 10,000 rpm (~13,000g). Upper phase was collected and centrifuged in a TH-641 rotor for 36,000 rpm (~222,000g) for 2.5 hr. The supernatant was removed and the pellet was dissolved in 100 μl of buffer phosphate 0.01M pH 7.0. The purified virus sample was subjected to Western blot analysis that showed the viral CP detected by a specific ToBRFV antiserum. The virus preparation was visualized by transmission electron microscopy scan.

### Transmission electron microscopy (TEM)

For TEM analysis, a sample of 5μL virus preparation was applied on 1% alcian blue treated, 300 mesh carbon-coated copper TEM grids. Samples were incubated for 10 minutes and then excess liquid was blotted. Samples were washed three times with distilled water, and stained with 1% Phosphotungstic acid. After air drying, samples were visualized using an FEI Tecnai T12 TEM operated at 120 kV and equipped with a Gatan ES500W Erlangshen camera. Scaling was done using a standard of known size measured at different magnifications. Viral length of each particle was measured by stretching a line from end to end.

#### Bioassay for ToBRFV particles isolated from contaminated hives

Comb extracts from contaminated and un-infected hives were suspended in 0.01M phosphate buffer pH 7.0 and applied on leaves of *N*. *tabacum* cv. Samsun and *N*. *rustica* plants. The plants were left to grow and symptom development was inspected.

#### Next generation sequencing (NGS) and bioinformatics analysis

A sampled bumblebee from beehive E was immersed in 0.01M phosphate buffer pH 7.0 for 5 min and the washing solution was collected. The bumblebee was then incubated for 30 min in a 0.1% hypochlorite solution and subsequently washed five times with water. The collected washing solution and the washed bumblebee were subjected to viral RNA extraction using Accuprep Viral RNA Extraction kit (Bioneer, Korea). The obtained RNA was used for the construction of TruSeq RNA sample prep kit (Illumina, San Diego, CA, USA) according to manufacturer protocol then the two libraries were sequenced using Illumina machine Hiseq 2500 as a single-end with 100 bases in read. For bioinformatics analysis the fastq raw data was subjected to quality filtering and adapter removal with the software trimmomatic [18]. Then we used Virusdetect software [19] to detect possible viruses in the sample using both a plant virus database and an invertebrate virus database.

## Results

### Studying the dispersal of ToBRFV during bumblebee buzz pollination

**Fig 1:**
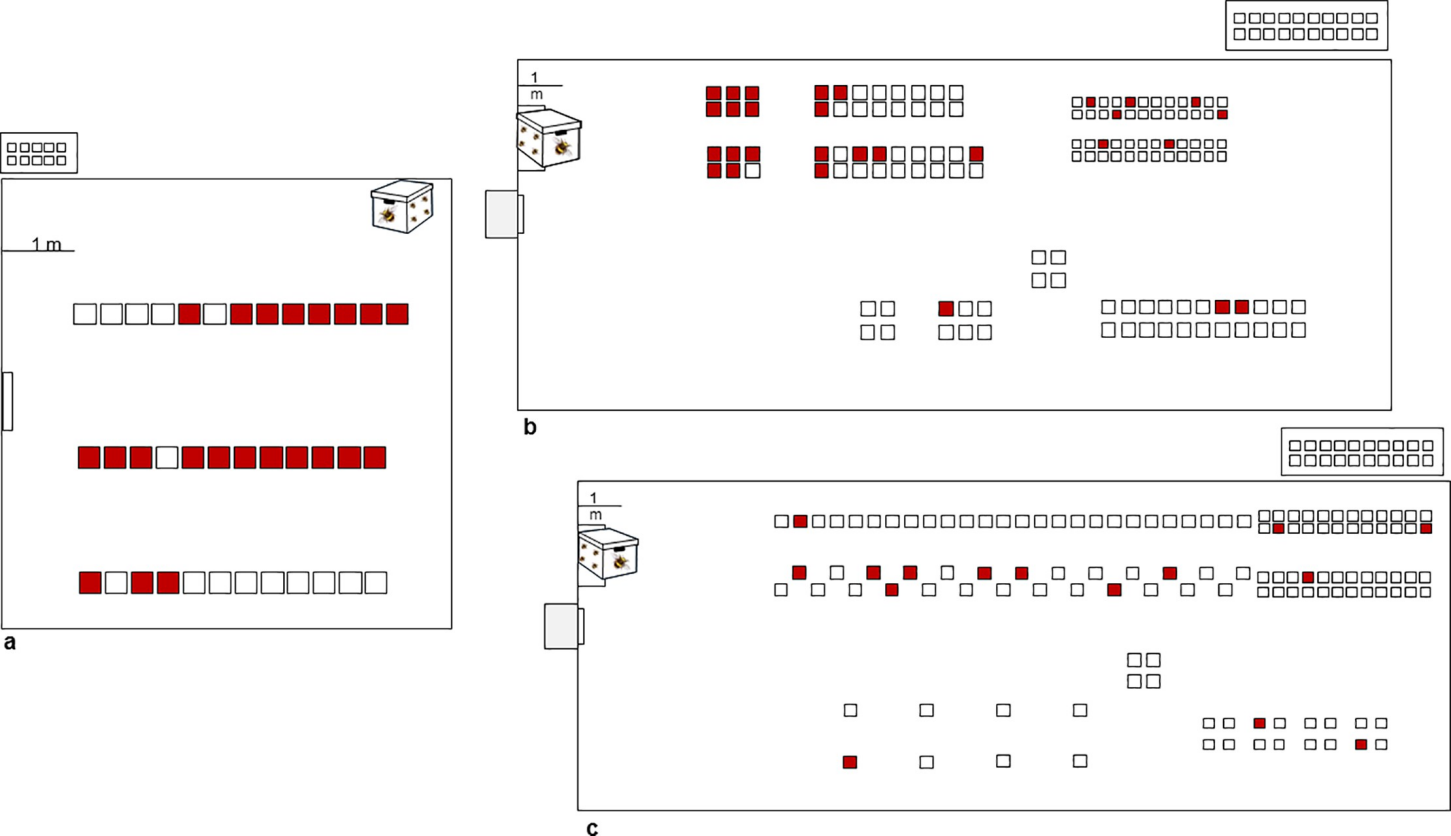
ToBRFV contaminated beehives spread viral disease. A schematic presentation summarizing the three glass-/net-house experiments using ToBRFV infected bumblebee hives. Adjacent to each experimental glass-/net-house is a depiction of a control experiment of tomato plants not exposed to the contaminated hives. **a.** A contaminated bumblebee hive inserted to a glasshouse of un-infected tomato plants cv. M82. **b.** A contaminated bumblebee hive inserted to a net-house of un-infected tomato plants cv. Ikram. **c.** A contaminated bumblebee hive inserted to a net-house of un-infected tomato plants cv. Ikram and Shiran. Red closed rectangles represent contaminated plants. Empty rectangles represent uninfected plants.

**Fig 2:**
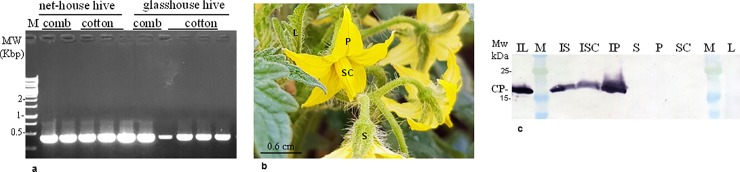
ToBRFV is present in hive components of beehives used in exp. # 1 and # 2 and in infected tomato flowers. **a.** Comb and cotton from contaminated beehives subjected to RT-PCR analysis using F-5931 and R-3’UTR primers. **b.** Dissected tissue parts of ToBRFV infected tomato plants. **c.** Western blot analysis showing coat protein of ToBRFV in dissected tomato flowers. I, ToBRFV infected plant; L, leaves; S, sepal; P, petal; SC, stamen encompassing the carpel; M, molecular size marker; CP, coat protein.

It has been shown that bumblebees may transfer the tobamovirus TMV between contaminated and adjacently planted uncontaminated tomato plants [15]. We therefore asked whether tobamoviruses adsorbed to the bumblebees might contaminate the hives and constitute a primary inoculum of the virus. This possibility was tested by inserting ‘used’ bumblebee hives that were previously placed in a ToBRFV infected greenhouse in HaBsor (Western Negev region), into glass-/net-houses containing only un-infected healthy tomato plants (Fig 1) while avoiding any possible mechanical transmission of the virus. In experiments #1, #2 and #3, leaf samples were collected from each plant on the 19^th^, 26^th^ and 38^th^ day post hive insertion, respectively. All plants were tested for the presence of ToBRFV using ELISA (Fig 1). The ELISA results are shown in Table 1. All samples that were ELISA positive for ToBRFV were analyzed by RT-PCR confirming the results. Apparently, the infection ratios of the tomato plants exposed to ToBRFV contaminated hives ranged between 11.7% and 59.5%. The low infection ratio occurred in Exp. #3, in which the bumblebees were rarely observed in foraging behavior. Experiment duration was determined by the presence of live bumblebees in the hives. At the end of the experiments, hive components from beehives of experiments # 1 and # 2, were analyzed for the presence of ToBRFV using RT-PCR analysis (Fig 2A). The results show an amplicon of ~460bp of the ToBRFV genome amplification at 3’ end of the virus genome. Western blot analysis of infected flowering tomato plants shows that the virus is present in all tissue parts of the plants (Fig 2B and 2C). High concentrations of the virus are present on the petals, which are in contact with the bumblebees. Importantly, no ToBRFV infection occurred in the control glass/net-house experiments where tomato seedlings from the same source were planted and were not exposed to the bumblebee hives but were left un-touched for the whole duration of the experiments (Fig 1).

**Table 1:** ToBRFV contaminated bumblebee hives carry a primary inoculum that infects tomato plants.

*Experiment No./date	Structure	Tomato variety	Beehive source/ days in field	Beehive treatment duration/ Bee activity	Contaminated plants (ratio)	ELISA O.D. range (3xNR)/ tTest**
**#1/ April (2017)**	Glasshouse	M82	HaBsor/ >14 days	19 days/ high	22/37 59.5%	0.09–2.00/ p = 1.55e-06
**#2/April (2017)**	Net-house	Ikram	HaBsor/ >14 days	26 days/ medium	29/130 22.3%	0.03–2.16/ p = 1.63e-05
**#3/ May (2017)**	Net-house	Shiran and Ikram	Ramat Negev/ >30 days	14 days/ very low	15/128 11.7%	0.09–2.86/ p = 0.005

*Experiments were carried out in Volcani Center. O.D., optical density; NR, negative reference; O.D. range included values that amounted to the numbers obtained above a minimum ratio of three times the NR

** tTest: The NR data and the O.D. data were analyzed in two sample tTest assuming unequal variances.

### ToBRFV is present in bumblebee hives collected from contaminated greenhouses

**Fig 3:**
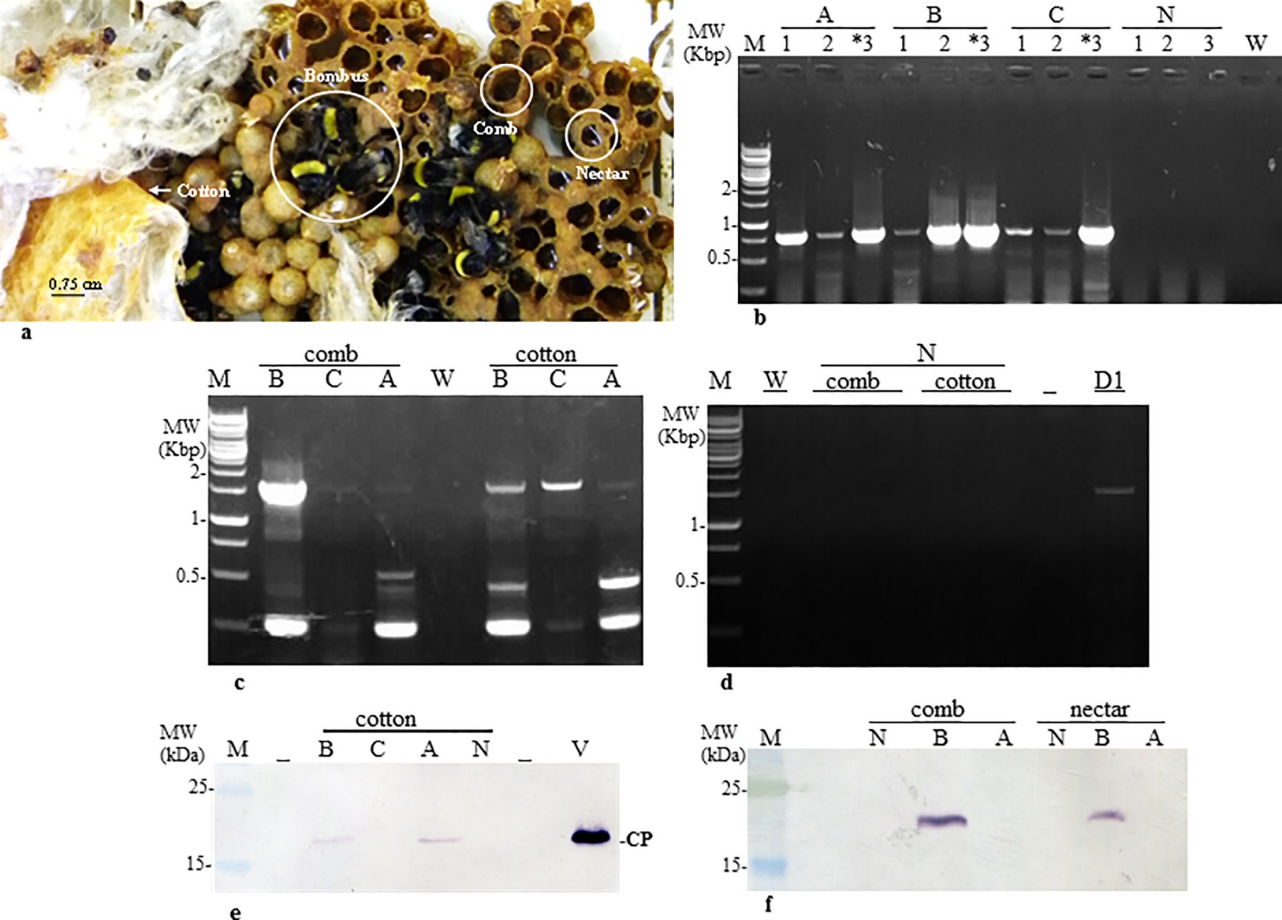
ToBRFV contaminates bumblebee hive components. **a.** Internal bumblebee hive components. **b.** ToBRFV detection in individual bumblebees from beehives A, B, C, N by RT-PCR analysis using F-5476 and R-6287 primers. **c.** ToBRFV detection in comb and cotton from beehives A-C by RT-PCR using F-1 and R-1572 primers. **d.** ToBRFV detection by RT-PCR in comb and cotton from a naive beehive using F-1 and R-1572 primers. **e,f.** Detection of ToBRFV coat protein in beehive components by Western blot analysis. N, naïve beehive; M, molecular size marker; W, water control; *, amplicon subjected to Sanger sequencing; D1, a bumblebee from contaminated beehive D; v, viral extraction; CP, coat protein.

Beehives were delivered from Ramat-Negev and were kept at -20°C for at least 24hr in order to stop beehive activity before sampling. The beehives A-C had 4, 10 and 12 bumblebees, respectively. The presence of ToBRFV was analysed in the bumblebees, in the enveloping-cotton, in the comb and in the nectar (Fig 3). A naive bumblebee hive served as a negative control (Fig 3D). Additional RT-PCR tests consistently showed that in total 4 out of 4 bees from hive A, 10 out of 10 bees from hive B and 10 out of 10 bees from hive C were positive for ToBRFV. One selected amplicon from each hive was subjected to Sanger sequencing. Importantly, the control naive bumblebees and the naive beehive were negative for the virus (Fig 3). The results indicate that beehives supplied from the companies were ToBRFV free, but only when placed in contaminated glasshouses the beehives were infected by ToBRFV.

**Fig 4:**
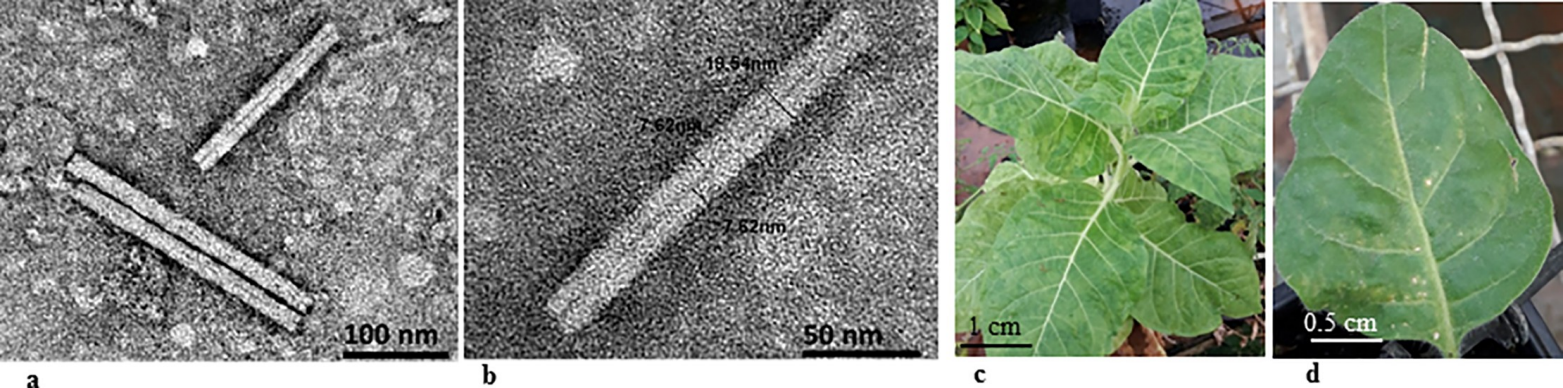
Electron micrograph of infectious tobamovirus particles purified from contaminated comb from bumblebee hive B. **a,b.** A typical tobamovirus particle morphology of Σ300 nm in length and Σ20 nm in width visualized by transmission electron microscopy. **c.** The pathogenicity of the purified virus preparation from bumblebee comb confirmed by bioassay on *Nicotiana tabacum* cv. Samsun showing typical mosaic and mottling symptoms. **d.** Bioassay of viral preparation from bumblebee comb shows necrotic local lesions on *N*. *rustica*.

Selected samples of the comb and cotton elements that were tested positive for the virus using RT-PCR, (Fig 3C), were also analysed by Western Blot (Fig 3E and 3F). Apparently, there are differences in sensitivity threshold of the detection method used, as reflected in the results obtained for the beehive components that were all positive for ToBRFV contamination by RT-PCR amplification assay but the majority of the samples were negative for the virus when analysed by Western blot analysis (Fig 3). In all Western blot analyses however, beehive B consistently showed positive results for a ToBRFV contamination of the comb (3 out of 3 tests), of the cotton (5 out of 5 tests) and also of the sugar water (nectar) (Fig 3F). Beehive B comb was therefore selected for virus purification followed by TEM analysis in order to verify the integrity of particle morphology (Fig 4A and 4B).

### Bioassay of ToBRFV purified from contaminated hives

Tobacco test plants: *N*. *tabacum* cv. Samsun and *N*. *rustica* were inoculated with ToBRFV particles purified from contaminated hives in order to validate the infectivity potential of the ToBRFV present in the contaminated hives. Mosaic and mottling symptoms indicating systemic infection were observed in *N*. *tabacum* cv. Samsun and typical necrotic local lesions were observed on *N*. *rustica* (Fig 4C and 4D). Leaf samples from symptomatic plants, analyzed by ELISA and RT-PCR, were positive for ToBRFV.

## ToBRFV is differentially detected in dissected bumblebees collected from contaminated hives

**Fig 5:**
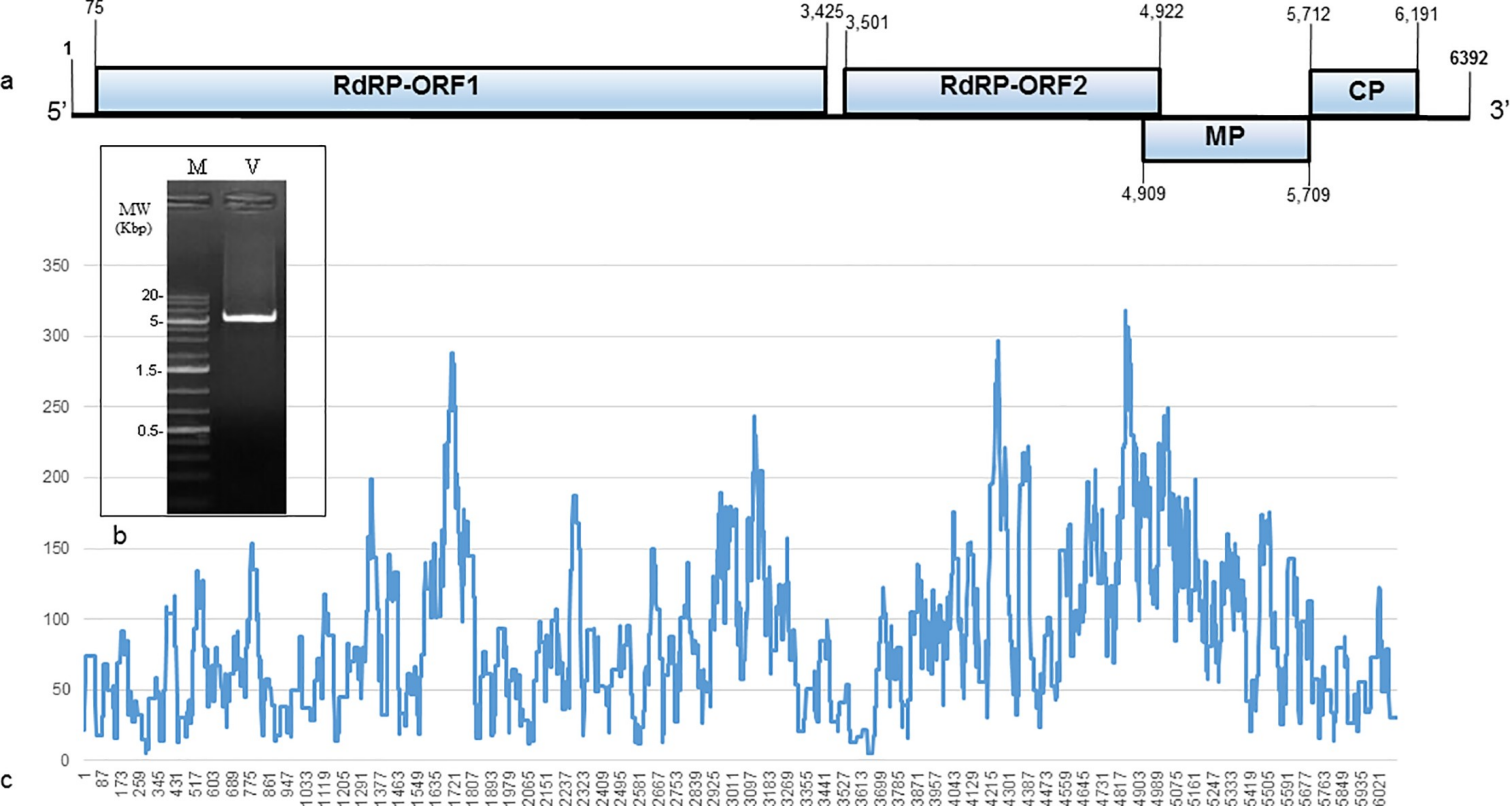
ToBRFV adhering to a bumblebee. **a.** A diagram of ToBRFV genome organization. **b.** RT-PCR of ToBRFV starting at position 264 to 5,531 covering ~82% of the genome. **c.** Distribution of NGS reads along the ToBRFV genome. RdRP, RNA dependent RNA polymerase; MP, movement protein; CP, coat protein; M, molecular size marker; V, RT-PCR amplification of ToBRFV genome.

**Fig 6:**
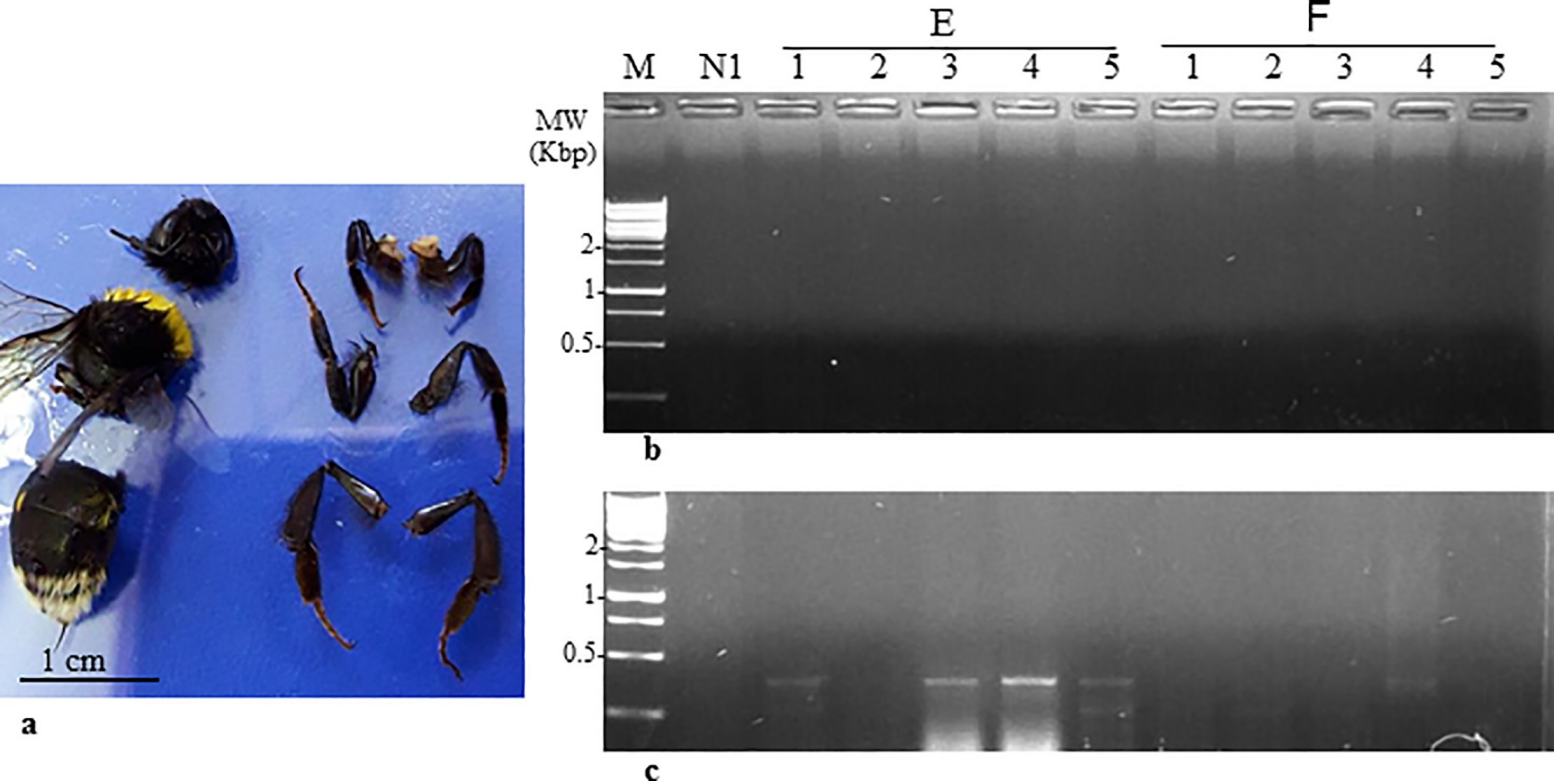
Detection of ToBRFV in dissected bumblebee body parts. **a.** A dissected bumblebee. **b.** Negative detection of ToBRFV in dissected bumblebee heads by RT-PCR using F-5,931 and R-6,307 primers. **c.** Positive detection of ToBRFV in dissected bumblebee abdomens by RT-PCR using F-5,931 and R-6,307 primers. E,F, contaminated bumblebee hives; N, naive bumblebee hive; M, molecular size marker.

A bumblebee from contaminated hive E was washed with 0.01M Phosphate buffer pH 7.0. The collected washing solution was analyzed for ToBRFV that adheres to the bee using RT-PCR followed by ToBRFV sequencing covering 5,267bp, and transcriptomic NGS (Illumina His-seq) (Fig 5). Bioinformatics analysis allowed to assemble the ToBRFV genome from the RNA extracted from the washing solution (Fig 5C) while no ToBRFV genome sequences were detected in the body extract of the washed bee. In addition, body parts of dissected bumblebees from infected and un-infected hives were analyzed for the presence of ToBRFV using RT-PCR (Fig 6B and 6C). The results obtained by the RT-PCR analysis showed positive detection of ToBRFV in the abdomens of bumblebees from contaminated hives (5/10), while the heads were negative for the virus (0/10).

## Discussion

Tobamoviruses are mechanically transmitted seed borne viruses that show very low seed transmission rate [20–23]. This suggests that fertilization process and embryonal contamination are not essential for tobamovirus spread. However, in a recent study we found that *Cucumber green mottle mosaic virus* (CGMMV) is spread from CGMMV-infected melon and cucumber plants to non-infected plants during honeybee *Apis mellifera* pollination in a greenhouse [24]. It was therefore suggested that the tobamovirus physically adsorbs to the honeybees and the virus is mechanically spread while the honeybees are foraging [24]. In tomato plants, bumblebees are more efficient than honeybees in their contribution to fruit production and quality [25] It has been reported that the bumblebee *Bombus terrestris* is able to transmit the TMV virus between infected and adjacently planted uninfected tomato plants [15]. The experiments were carried out in greenhouses already containing a primary inoculum of TMV in the plants. Importantly, the TMV-contaminated bumblebees were not successful in transmitting the virus to healthy un-infected tomato plants grown in a greenhouse separate from TMV infected tomato plants [15]. The data in that report implies that in the bumblebee hives there is no preservation of infectious TMV viral particles. We wanted to find out whether this conclusion is true for a greenhouse infected bumblebee hives. In our current study, we therefore employed a different strategy. We placed ToBRFV-contaminated-beehives, which were previously positioned in a virus-contaminated greenhouse, in our experimental glass-/net-houses containing only un-infected healthy tomato plants. In these designed experiments we prevented ToBRFV contamination *via* biting insects and small animals (e.g. mice). Primarily we asked whether infectious ToBRFV particles are preserved in the beehives and might constitute a primary inoculum transmitted by the bumblebees. In our designed experiments we are also avoiding any alterations in the bumblebee foraging behavior that may result from possible preference of the bumblebees to visit infected tomato plants versus the un-infected plants, as was observed in CMV-infected tomato plants [26]. Under the latter circumstances, in a greenhouse that has both infected and un-infected tomato plants the bumblebees may revisit the virus infected plants and thereby reduce visits to the un-infected plants. Thus, the apparent virus spread might not reflect the full potential of the hive to disperse the virus. We therefore analyzed the spreading potential of ToBRFV-contaminated hives in glass-/net-houses containing only un-infected tomato plants. Indeed, we observed that the bumblebees that were inserted into the un-infected-glass-/net-house were transmitting ToBRFV (Fig 1 and Table 1). However, a disadvantage of our designed experiments that were based on greenhouse-contaminated-ToBRFV hives was the demonstration of high variation between contaminated hives, as reflected in the varied ratio of infected plants versus un-infected plants between experiments. This may be the result of bumblebee vitality/activity that was accordingly modified during the experiments. Importantly, the pattern of the infection was sporadic which is consistent with our care to prevent any mechanical transmission of the virus by any other means than *via* the bumblebees. This was also confirmed by the control experiments that were negative for ToBRFV.

Similar to CGMMV transmission by honeybees [24] ToBRFV may physically adhere to the pollen grains attached to the bumblebees. The bumblebees may transmit the virus by transferring crud sap using their mandibles, or mechanically through their vibrating bodies. Based on our results, we suggest that during pollination, vibrations of the bumblebees may contribute to mechanical transmission of the virus. This is supported by the pollination during the biological assay and by transcriptome analysis of RNA adhering to a bumblebee that retrieved the viral genome sequence in the washing solution and non in the extraction of the washed bumblebee. Furthermore, dissection of the bumblebees showed that the bumblebee heads were devoid of the virus while the abdomens were highly contaminated by the virus (Fig 6B and 6C).

The contribution of bumblebees to tomato pollination is not-expendable. However, bumblebee hives might carry an infectious primary inoculum of viruses contaminating a greenhouse. According to our current study viruses in a bumblebee hive may be indicative of the plant viruses infecting a growing area and the bumblebees may therefore be subjected to viral metagenomics studies done on beneficial pollinators [27–29]. This study also emphasizes the necessity to renew practical maneuvers in tomato growth practices and use more often naive bumblebee hives before planting the following growth cycle since virus contaminated bumblebee hives might carry a primary inoculum of ToBRFV.
